# Persistent MRI Findings Unique to Blast and Repetitive Mild TBI: Analysis of the CENC/LIMBIC Cohort Injury Characteristics

**DOI:** 10.1093/milmed/usae031

**Published:** 2024-02-24

**Authors:** David F Tate, Benjamin S C Wade, Carmen S Velez, Erin D Bigler, Nicholas D Davenport, Emily L Dennis, Carrie Esopenko, Sidney R Hinds, Jacob Kean, Eamonn Kennedy, Kimbra Kenney, Andrew R Mayer, Mary R Newsome, Carissa L Philippi, Mary J Pugh, Randall S Scheibel, Brian A Taylor, Maya Troyanskaya, John K Werner, Gerald E York, William Walker, Elisabeth A Wilde

**Affiliations:** Department of Neurology, Traumatic Brain Injury and Concussion Center, University of Utah, Salt Lake City, UT 84132, USA; George E. Wahlen VA Salt Lake City Healthcare System, Salt Lake City, UT 84148, USA; Department of Psychology, Brigham Young University, Provo, UT 84604, USA; Department of Neurology, Traumatic Brain Injury and Concussion Center, University of Utah, Salt Lake City, UT 84132, USA; Massachusetts General Hospital, Harvard Medical School, Boston, MA 02114, USA; Department of Neurology, Traumatic Brain Injury and Concussion Center, University of Utah, Salt Lake City, UT 84132, USA; George E. Wahlen VA Salt Lake City Healthcare System, Salt Lake City, UT 84148, USA; Department of Neurology, Traumatic Brain Injury and Concussion Center, University of Utah, Salt Lake City, UT 84132, USA; Department of Psychology, Brigham Young University, Provo, UT 84604, USA; Departments of Neuroscience, Brigham Young University, Provo, UT 84604, USA; Minneapolis Veterans Affairs Health Care System, Minneapolis, MN 55417, USA; Department of Psychiatry and Behavioral Sciences, University of Minnesota, Minneapolis, MN 55454, USA; Department of Neurology, Traumatic Brain Injury and Concussion Center, University of Utah, Salt Lake City, UT 84132, USA; George E. Wahlen VA Salt Lake City Healthcare System, Salt Lake City, UT 84148, USA; Department of Rehabilitation and Human Performance, Icahn School of Medicine at Mount Sinai, New York, NY 10029, USA; Department of Neurology, Uniformed Services University, Bethesda, MD 20814, USA; George E. Wahlen VA Salt Lake City Healthcare System, Salt Lake City, UT 84148, USA; Department of Population Health Sciences, University of Utah, Salt Lake City, UT 84108, USA; Department of Population Health Sciences, University of Utah, Salt Lake City, UT 84108, USA; Department of Neurology, Uniformed Services University, Bethesda, MD 20814, USA; National Intrepid Center of Excellence, Walter Reed National Military Medical Center, Bethesda, MD 20814, USA; The Mind Research Network, University of New Mexico Health Science Center, Albuquerque, NM 87106, USA; Michael E. DeBakey Veterans Affairs Medical Center, Houston, TX 77030, USA; H. Ben Taub Department of Physical Medicine & Rehabilitation, Baylor College of Medicine, Houston, TX 77030, USA; Department of Psychological Sciences, University of Missouri-St. Louis, St. Louis, MO 63121, St. Louis; George E. Wahlen VA Salt Lake City Healthcare System, Salt Lake City, UT 84148, USA; Department of Population Health Sciences, University of Utah, Salt Lake City, UT 84108, USA; Michael E. DeBakey Veterans Affairs Medical Center, Houston, TX 77030, USA; H. Ben Taub Department of Physical Medicine & Rehabilitation, Baylor College of Medicine, Houston, TX 77030, USA; Department of Imaging Physics, The University of Texas M. D. Anderson Cancer Center, Houston, TX 77030, USA; Michael E. DeBakey Veterans Affairs Medical Center, Houston, TX 77030, USA; H. Ben Taub Department of Physical Medicine & Rehabilitation, Baylor College of Medicine, Houston, TX 77030, USA; Department of Neurology, Uniformed Services University, Bethesda, MD 20814, USA; Imaging Associates of Alaska, Anchorage, AK 99508, USA; Department of Physical Medicine and Rehabilitation, Virginia Commonwealth University, Richmond, VA 23298, USA; Department of Neurology, Traumatic Brain Injury and Concussion Center, University of Utah, Salt Lake City, UT 84132, USA; George E. Wahlen VA Salt Lake City Healthcare System, Salt Lake City, UT 84148, USA; Michael E. DeBakey Veterans Affairs Medical Center, Houston, TX 77030, USA; H. Ben Taub Department of Physical Medicine & Rehabilitation, Baylor College of Medicine, Houston, TX 77030, USA

## Abstract

**Introduction:**

MRI represents one of the clinical tools at the forefront of research efforts aimed at identifying diagnostic and prognostic biomarkers following traumatic brain injury (TBI). Both volumetric and diffusion MRI findings in mild TBI (mTBI) are mixed, making the findings difficult to interpret. As such, additional research is needed to continue to elucidate the relationship between the clinical features of mTBI and quantitative MRI measurements.

**Material and Methods:**

Volumetric and diffusion imaging data in a sample of 976 veterans and service members from the Chronic Effects of Neurotrauma Consortium and now the Long-Term Impact of Military-Relevant Brain Injury Consortium observational study of the late effects of mTBI in combat with and without a history of mTBI were examined. A series of regression models with link functions appropriate for the model outcome were used to evaluate the relationships among imaging measures and clinical features of mTBI. Each model included acquisition site, participant sex, and age as covariates. Separate regression models were fit for each region of interest where said region was a predictor.

**Results:**

After controlling for multiple comparisons, no significant main effect was noted for comparisons between veterans and service members with and without a history of mTBI. However, blast-related mTBI were associated with volumetric reductions of several subregions of the corpus callosum compared to non–blast-related mTBI. Several volumetric (i.e., hippocampal subfields, etc.) and diffusion (i.e., corona radiata, superior longitudinal fasciculus, etc.) MRI findings were noted to be associated with an increased number of repetitive mTBIs versus.

**Conclusions:**

In deployment-related mTBI, significant findings in this cohort were only observed when considering mTBI sub-groups (blast mechanism and total number/dose). Simply comparing healthy controls and those with a positive mTBI history is likely an oversimplification that may lead to non-significant findings, even in consortium analyses.

## INTRODUCTION

Mild traumatic brain injury (mTBI) remains a significant public health concern that poses several unique challenges to clinicians and researchers.^1^ First, since the diagnosis of mTBI is based on clinical criteria, absent of any objective biomarker of injury, it can be difficult to diagnose. This is especially true when injuries are temporally remote.^2^ Second, even though about 20 to 30% of patients with an mTBI may develop persistent symptoms,^3^ accurately identifying those at risk for chronic problems following a mTBI is challenging.^4,5^ Third, the mechanism and location of injury can vary across and within samples contributing potential, unknown variance to outcomes.^6^ For these reasons, there has been a significant endeavor to identify consistent diagnostic and prognostic biomarkers that would improve diagnosis, monitor recovery or progression of symptoms, and evaluate potential treatment effects (pharmacodynamic biomarkers).

MRI represents one of the clinical tools at the forefront of research efforts aimed at identifying more objective biomarkers of injury. Successful identification of accurate MRI biomarkers could be used to support clinical care (e.g., patient specific cognitive rehabilitation planning) in this functionally heterogeneous population.^7,8^ As such, volumetric MRI has been successfully used to characterize structural abnormalities and their association with neurobehavioral and neurocognitive outcomes in patients with TBI, especially those with moderate-to-severe injury.^8^ Nonetheless, even in mTBI cases, some consistent findings are emerging, such as volumetric and shape differences, especially within the frontal and temporal lobes and in subcortical nuclei like the thalamus, hippocampus, caudate, and nucleus accumbens.^9–14^ Diffusion MRI has also demonstrated several important and significant findings in mTBI that are related to key clinical TBI features.^15,16^ However, for both the volumetric and diffusion MRI literature, there are also non-significant, equivocal, or possibly unexpected (i.e., increased volumes or scalar metrics) findings^17–19^ that are difficult to interpret and require further investigation. There have been many reasons postulated for these equivocal findings including but not limited to methodological difference (i.e., MRI site variability in acquisition and variable measurement tools) and/or sample differences (i.e., sample size and demographic differences). Regardless, the simple fact of the matter is that the MRI findings in mTBI remain complicated.

Another possible factor impacting the variability of findings is the fact that not only do many of these studies examine MRI sequences independently, but they also tend to focus on a limited number of regions of interest (ROIs). Recent studies looking at methods to integrate the metrics of different sequences are beginning to demonstrate the improved sensitivity of MRI to mTBI diagnosis. However, there is still a need for studies of sufficient sample size that describe the relationships between different clinical features of the injury (i.e., loss of consciousness [LOC], number of TBIs, etc.) and different MRI sequence metrics or more broad brain-wide ROIs. The original Chronic Effects of Neurotrauma Consortium (CENC)^20^ prospective longitudinal study (PLS), now under the Long-Term Impact of Military-Relevant Brain Injury Consortium (LIMBIC), is one of the few observational studies of the late effects of mTBI in military medicine that has or will have sufficient sample sizes to examine multiple ROIs across multiple MRI metrics. This type of analysis might be important as it might improve the sensitivity of MRI as a diagnostic and prognostic biomarker or help identify relationships not frequently reported in the literature. The LIMBIC-CENC PLS originally enrolled current and former service members (SMs) with a range of mTBI exposures and a history of combat deployment in Operation Iraqi Freedom, Operation Enduring Freedom, and Operation New Dawn. Under LIMBIC, it began including SMs from all eras of combat deployment and as such has established a cohort of combat veterans and SMs with well-characterized mTBI histories that are followed longitudinally using symptom, biological, and performance measures. An important aspect of the LIMBIC-CENC project is the sample size. Because of sample size restrictions, previous mTBI imaging studies have been limited in the number of variables that can be examined and as such may underestimate the obvious heterogeneous and multifaceted nature of mTBI.

In this analysis of available cross-sectional baseline study data, we conduct an examination of the associations between MRI volumetric/diffusion measures and several clinical features associated with TBI injury history from LIMBIC-CENC PLS cohorts of military-related mTBI. More specifically, the purpose of this study is to directly examine group differences for several mTBI history characteristics (i.e., LOC, post-traumatic amnesia [PTA], number of mTBI(s), mechanism [blast related vs. non-blast related]) and quantitative MRI measurements in a large sample of participants from the LIMBIC-CENC PLS. We hypothesized that these clinical features of mTBI would be associated with overlapping yet unique differences in MRI-derived brain metrics.

## MATERIALS AND METHODS

### Participants

Participants in this study include the current and former veterans and SMs enrolled as study participants in the LIMBIC-CENC PLS with complete volumetric and diffusion MRI data. Participants included those recruited and enrolled at eight sites across the USA (Table I). A total of 976 participants with complete and verified study entry (baseline) clinical and MRI data were included in the final analyses.

**TABLE I.** Basic Demographic Information for Each Site

Site	Total (*N*)	Age (SD)	Male (*n*)	mTBI with LOC	mTBI with PTA	mTBI positive	Primary blast related	Median and maximum number of mTBI exposures	PCL5 mean (SD)	PHQ9 mean (SD)
Total	976	40.3 (9.86)	841	525	578	794	355	2 (15)	26.7 (19.3)	8.11 (6.11)
1	258	44.6 (9.66)	212	115	136	197	80	1 (9)	24.4 (19.8)	7.28 (6.31)
2	166	34.0 (7.33)	150	108	112	133	72	2 (8)	33.4 (19.8)	9.84 (6.05)
3	175	38.6 (9.22)	151	93	103	138	63	2 (11)	28.3 (17.5)	8.23 (5.13)
4	118	41.9 (10.7)	101	71	69	106	54	2 (12)	31.1 (20.4)	9.55 (7.22)
5	64	40.9 (8.47)	55	44	48	63	33	3 (15)	30.5 (19.2)	9.92 (5.76)
6	93	40.2 (9.61)	79	41	50	78	23	2 (12)	16.3 (14.6)	5.73 (5.60)
7	49	41.2 (10.1)	45	21	25	32	10	1 (7)	18.5 (16.8)	5.78 (5.52)
8	53	37.5 (7.83)	48	32	35	47	20	2 (7)	23.9 (14.4)	7.23 (4.53)

### Inclusion and Exclusion Criteria

The inclusion criteria were as follows: (1) Age ≥18 years; (2) history of at least one combat deployment; (3) score of >1 on at least one item on the Deployment Risk and Resilience Inventory-2.^21^ Individuals with severe psychiatric (e.g., schizophrenia) and neurological (e.g., stroke) disorders were not enrolled. Basic demographic and clinical features are summarized in Tables I and II along with the sample sizes included in the final analyses. Each participant underwent a detailed semi-structured interview (called potential concussive event [PCE] mapping) to identify all lifetime PCEs that are then subjected to a validated mTBI diagnostic algorithm. This interview and TBI determination process was previously described in more detail.^22,23^ Briefly, this instrument was designed to obtain not only the frequency of events meeting diagnostic criteria for mTBI but to assess attributes of each mTBI through a series of standard follow-up questions about LOC, PTA, and other associated symptoms. The PCE interview also identifies a “signature” injury event as the worst PCE during combat deployment (or after combat deployment in some cases). Time since injury (TSI) is calculated from the signature injury or one that produced the most or most severe initial effects and, as such, may not be the most recent injury.^20^ Using data from the PCE mapping, the following groups were identified for comparison.

**TABLE II.** Observed Associations Between mTBI Classifications and Imaging Measures

Contrast	Region	B	Z	*P*
TBI history	rh_parstriangularis_thickness	0.30	3.20	<.01
	rh_rostralmiddlefrontal_thickness	0.29	3.31	<.01
Blast Status	CC_Posterior	−0.17	−2.16	<.05
	CC_Mid_Posterior	−0.20	−2.55	<.05
	CC_Central	−0.22	−2.71	<.01
	CC_Mid_Anterior	−0.25	−3.10	<.01
	SFO_AD	0.30	3.19	<.01
mTBI count	lh_presubiculum_body	0.07	2.84	<.01
	lh_subiculum_body	0.08	3.21	<.01
	lh_CA1_head	0.07	2.77	<.01
	lh_molecular_layer_HP_head	0.08	3.14	<.01
	lh_molecular_layer_HP_body	0.06	2.54	<.05
	lh_GC_ML_DG_head	0.08	3.05	<.01
	lh_CA4_head	0.08	3.03	<.01
	lh_CA3_head	0.06	2.54	<.05
	lh_Whole_hippocampal_body	0.07	2.85	<.01
	lh_Whole_hippocampal_head	0.08	3.03	<.01
	lh_Whole_hippocampus	0.07	2.60	<.01
	rh_transversetemporal_thickness	0.11	4.10	<.01
	rh_insula_thickness	0.08	3.10	<.01
	CGH_R_RD	0.10	3.64	<.01
	SCC_RD	0.09	3.06	<.01
	CGH_RD	0.10	3.76	<.01
	ACR_R_AD	0.07	2.68	<.01
	CGH_R_AD	0.13	4.64	<.01
	CR_R_AD	0.11	4.07	<.01
	PCR_R_AD	0.09	3.54	<.01
	SCR_R_AD	0.12	4.51	<.01
	IC_R_AD	0.07	2.62	<.01
	PLIC_R_AD	0.08	2.77	<.01
	RLIC_R_AD	0.08	2.90	<.01
	SLF_R_AD	0.07	2.50	<.05
	ACR_L_AD	0.07	2.69	<.01
	CGH_L_AD	0.08	2.98	<.01
	CR_L_AD	0.08	3.11	<.01
	PCR_L_AD	0.07	2.60	<.01
	SCR_L_AD	0.09	3.35	<.01
	IC_L_AD	0.06	2.29	<.05
	PLIC_L_AD	0.06	2.31	<.05
	RLIC_L_AD	0.07	2.57	<.05
	IFO_L_AD	0.09	3.20	<.01
	SS_L_AD	0.07	2.59	<.01
	ACR_AD	0.07	2.73	<.01
	Average_AD	0.07	2.41	<.05
	GCC_AD	0.07	2.43	<.05
	CGH_AD	0.10	3.74	<.01
	CR_AD	0.10	3.70	<.01
	PCR_AD	0.08	3.18	<.01
	SCR_AD	0.11	3.92	<.01
	IC_AD	0.07	2.48	<0.05
	PLIC_AD	0.07	2.63	<.01
	RLIC_AD	0.08	2.79	<.01
	IFO_AD	0.07	2.65	<.01
	SLF_AD	0.07	2.33	<.05
	ACR_R_MD	0.06	2.26	<.05
	ALIC_R_MD	0.06	2.41	<.05
	CGH_R_MD	0.12	4.55	<.01
	CR_R_MD	0.09	3.18	<.01
	PCR_R_MD	0.08	2.74	<.01
	SCR_R_MD	0.09	3.43	<.01
	CST_R_MD	0.08	2.83	<.01
	FX_ST_R_MD	0.06	2.19	<0.05
	IC_R_MD	0.08	2.96	<.01
	PLIC_R_MD	0.08	2.80	<.01
	RLIC_R_MD	0.13	4.56	<.01
	IFO_R_MD	0.08	2.86	<.01
	SFO_R_MD	0.07	2.44	<.05
	SLF_R_MD	0.08	2.77	<.01
	SS_R_MD	0.07	2.48	<.05
	ACR_L_MD	0.07	2.60	<.01
	CGH_L_MD	0.08	2.90	<.01
	CR_L_MD	0.08	2.89	<.01
	SCR_L_MD	0.07	2.53	<.05
	RLIC_L_MD	0.07	2.43	<.05
	IFO_L_MD	0.07	2.47	<.05
	SFO_L_MD	0.06	2.27	<.05
	SLF_L_MD	0.07	2.67	<.01
	SS_L_MD	0.06	2.38	<.05
	UNC_L_MD	0.07	2.60	<.01
	ACR_MD	0.07	2.41	<.05
	Average_MD	0.07	2.25	<.05
	BCC_MD	0.06	2.20	<.05
	CC_MD	0.07	2.59	<.01
	GCC_MD	0.07	2.35	<.05
	SCC_MD	0.11	3.79	<.01
	CGH_MD	0.11	3.96	<.01
	CR_MD	0.08	2.95	<.01
	PCR_MD	0.07	2.64	<.01
	SCR_MD	0.09	3.12	<.01
	CST_MD	0.07	2.46	<.05
	EC_MD	0.07	2.53	<.05
	IC_MD	0.07	2.63	<.01
	PLIC_MD	0.07	2.53	<.05
	RLIC_MD	0.11	4.30	<.01
	IFO_MD	0.08	2.68	<.01
	SFO_MD	0.08	3.03	<.01
	SLF_MD	0.08	2.82	<.01
	SS_MD	0.06	2.19	<.05

#### mTBI Group

Veterans and SMs with a positive history of mTBI (regardless of number of TBIs in history) were identified and compared to those with an entirely negative lifetime history of mTBI. The diagnosis of mTBI was made per Veterans Affairs/DoD diagnostic criteria using the PCE-structured interview, medical record review, and expert consensus by a central committee when diagnostic determination was questionable.

#### LOC Group

Veterans and SMs with mTBI history were also grouped into those reporting LOC less than 30 minutes during their signature injury event and those not reporting a LOC during the signature event. LOC has been identified as being associated with worse functional and imaging outcomes^24–26^ so this larger grouping is examined to determine what effect LOC has on volumetric and diffusion MRI metrics.

#### PTA Group

Veterans and SMs with mTBI history were also grouped into those reporting any PTA less than 24 hours following their signature injury event and those not reporting PTA. PTA has been identified as being associated with worse functional and imaging outcomes^27,28^ and is included as a grouping of interest to determine what effect PTA might have on imaging metrics.

#### TBI Count

The total number of lifetime mTBIs diagnosed for each patient was included as a raw count. Analysis of mTBI count was intended to examine the relation between the number of concussive events and imaging findings as there have been a number of studies that show a relationship between the total TBI number (dose) and worsening symptoms or imaging findings.^29,30^

#### Blast related vs. non-blast related

Veterans and SMs with an mTBI history were also grouped into any mTBI associated with a blast event. The designation of blast-related was given to individuals who identified their signature injury as being primarily related to blast exposure. Analyses of these groups were intended to identify potential imaging differences that might be associated with blast-related injuries in this cohort as blast-related injury has been an intense topic of investigation for veterans and SMs^31^ since this type of TBI exposure may differ pathophysiologically from other mechanisms of injury.^32^

#### Imaging acquisition and processing

Each participant underwent MRI assessment using a standardized imaging protocol monitored at a central site for scan parameter compliance. Both the T1-weighted and diffusion MRI scans were utilized in this group of analyses. Even though scan parameters at each site were designed to be similar (e.g., Alzhiemers Disease NeuroImaging network model), it should be noted that scan parameters did vary minimally at each site and sites 7 and 8 were excluded from diffusion analyses reported here because of a lack of B0 images.

### Image Post-Processing

After visual inspection for quality control, each imaging data set was processed at a single site (University of Utah) to ensure standardization of processing. All the volumetric measures were derived using the FreeSurfer processing pipelines that have been described in detail elsewhere and used in many TBI studies.^18,33,34^ These methods utilized the T1-weighted images to derive quantitative measures (volume and thickness) for cortical and subcortical structures using the “recon-all” command and associated processing algorithm. This command initializes a pipeline of sequential procedures that result in volumetric output for both gray and white matter (WM) structures in the brain. These steps include skull stripping, inhomogeneity correction, segmentation of tissue classes, and probabilistic mapping and labeling of cortical and subcortical structures.

The diffusion MRI sequence data were also processed at a single site using standardized processing procedures developed by the Enhancing NeuroImaging Genetics through Meta-Analysis Diffusion Tensor Imaging Working Group.^35^ Diffusion MRI data underwent several preprocessing steps including eddy current correction, brain extraction, and tensor fitting. Using the fMRI Software Library tract-based spatial statistics tools using predefined Enhancing NeuroImaging Genetics through Meta-Analysis templates and atlases, the scalar metrics for 24 ROIs (Johns Hopkins University WM atlas) were extracted.^36^

#### Statistical procedures

A series of regression models with link functions appropriate for the model outcome were used to evaluate the relationships between imaging measures and clinical features of mTBI. Each model included acquisition site, participant sex, age, time since signature injury (defined by the subject as the worst mTBI event during deployment, but if no deployment events were mTBIs, then time since the worst post-deployment mTBI was used), PTSD (captured by the Post-traumatic Stress Disorder Checklist for DMS-5), and depressive symptoms (captured by the Patient Health Questionnaire 9) as covariates. Separate regression models were fit for each ROI where said region was a predictor. Binary logistic regression was used to model associations between regions and mTBI status or blast status as these were binary outcomes. Associations with LOC status and PTA status were modeled using analysis of covvariance models as each had three levels with no assumed ordering. TBI count was modeled using zero-inflated Poisson regression since the outcome was a count variable and a history of no TBIs (coded as 0) is presumed to disproportionately impact the distribution when including those with a history of one or more mTBIs. Continuous predictors and covariates (imaging measures, age, TSI, Post-traumatic Stress Disorder Checklist for DMS-5 score, and Patient Health Questionnaire 9 score) were scaled and zero centered. Rosner’s test for outliers was applied to detect and exclude up to 10 outliers for each ROI value. Rosner’s test is used to avoid the problem of masking in which multiple outliers of similar values can go undetected. Post-hoc tests exploring participant sex as a moderator of associations between regional measures and mTBI clinical features were conducted by repeating the aforementioned models with an additional region-by-sex interaction term. We applied an false discovery rate adjustment separately to the set of *P*-values derived from each of the four outcomes partitioned by brain hemisphere (left, right, midline, or whole brain). All analyses were conducted using R version 3.6.2 (open source).

Quantification of neuroimaging measures is commonly biased by idiosyncrasies of the scanner. Thus, in a multisite framework, it is important to account for systematic deviations across sites. To contend with this, we used the ComBat harmonization algorithm^37^ to harmonize estimations of cortical thickness, subcortical volume, and diffusion measures before final statistical analyses.

## RESULTS

### Clinical and Demographic Effects

A higher proportion of male participants had a positive history of mTBI compared to females (${\chi ^2}$ = 9.07, df = 1, *P* < .05). As expected, mTBI count (${\chi ^2}$ = 12.59, df = 2, *P* < .05), blast status (${\chi ^2}$ = 22.60, df = 1, *P* < .05), LOC status (${\chi ^2}$ = 12.98, df = 2, *P* < .05), and PTA status (${\chi ^2}$ = 10.65, df = 2, *P* < .05) were disproportionately associated with males relative to females. Participants with histories of non-blast mTBIs were significantly older than those with blast-related mTBIs (t = 2.38, df = 734.59, *P* < 0.05); however, age was not significantly associated with mTBI status, mTBI count, LOC status, or PTA status.

**FIGURE 1.**
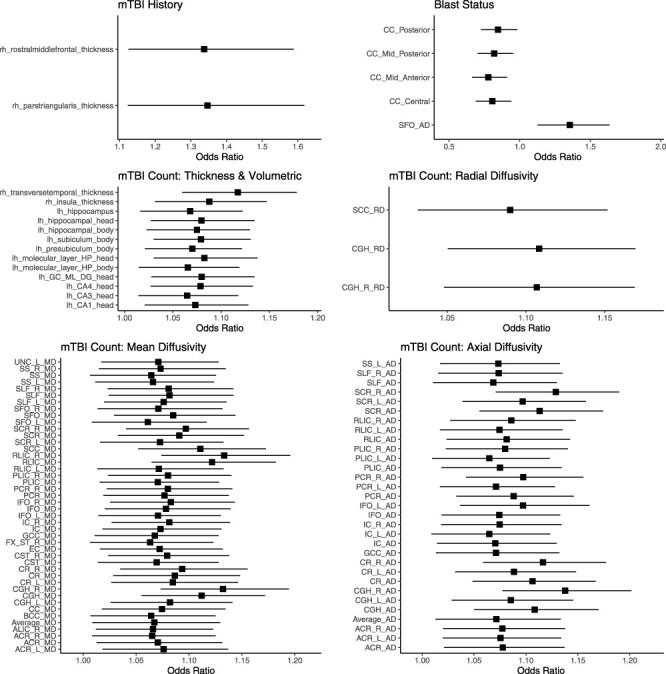
Forrest plots showing the odds ratios for volumetric and diffusion brain measures and various clinical measures associated with TBI.

Significant results after controlling for multiple comparison described later are summarized in Table II and illustrated in Fig. 1.

### Association with mTBI History

Participants with a history of mTBI had increased thickness of the right pars triangularis (B = 0.29, z = 3.19, *P* < .01) and the right rostral middle frontal gyrus (B = 0.29, z = 3.31, *P* < .01) when compared to those without a history of mTBI.

### Associations with Blast Status

Blast-related brain injuries were associated with volumetric reductions of several subregions of the corpus callosum after controlling for multiple comparisons: The posterior (B = −0.16, z = −2.15), mid-posterior (B = −0.19, z = −2.54, *P* < .05), central (B = −0.21, z = −2.70, *P* < .01), and mid-anterior (B = −0.25, z = −3.10, *P* < .01) segments. The axial diffusivity of the superior fronto-occipital (SFO) fasciculus was elevated in participants with blast-related brain injuries (B = 0.3, z = 3.19, *P* < .01).

### Associations with TBI Count

Several hippocampal subfields, cortical areas, and WM tracts remained significantly associated with total TBI count following adjustment for multiple comparisons. Increased mTBI counts were associated with increased volumes of the left hippocampal body (B = 0.07, z = 2.84, *P* < .01), left hippocampal head (B = 0.07, z = 3.02, *P* < .01), and whole hippocampus (B = 0.06, z = 2.59, *P* < .01). Similarly, increased mTBI counts were associated with increased cortical thickness of the right transverse temporal gyrus (B = 0.11, z = 4.09, *P* < .01) and right insula (B = 0.08, z = 3.10, *P* < .01). Additionally, widespread associations between increased mTBI counts and elevated mean diffusivity, axial diffusivity, and radial diffusivity were observed including anterior, posterior, and superior subregions of the corona radiata; internal, external, posterior, and retrolenticular portion of the internal capsule; and association fibers like the superior longitudinal fasciculus and inferior fronto-occipital fasciculus.

Post-hoc analyses identified that widespread associations between mTBI count and imaging measures were moderated by sex. In particular, a collection of frontoparietal regions were thicker in female participants with higher mTBI counts compared to male participants. Similarly, female SMs and veterans exhibited increased fractional anisotropy of certain WM tracts at higher mTBI counts. These significant interactions are detailed in Supplementary Table S1.

## DISCUSSION

In this large cohort of current and former SMs, we examined the impact of various clinical injury features on both volumetric and diffusion imaging measures with an average of 8 years from their signature mTBI. A simple comparison of veterans and SMs with and without a history of mTBI controlling for age, sex, acquisition site, time since signature injury, PTSD, and depressive symptoms revealed only sparse differences in regional cortical thickness estimates. However, certain features of mTBI history including the total number of lifetime mTBIs and mechanisms (i.e., blast related) of mTBI were significantly associated with more widespread MRI volumetric and diffusion MRI measurements. More specifically, when blast exposure was reported as the main “cause” of the signature mTBI, significant reductions in the central WM volumes (sections of the corpus callosum) along with the WM measures in the SFO fasciculus are noted. When the total number of mTBI exposures (regardless of etiology) was considered, there were many significant diffusion and volumetric associations noted as the number of mTBIs experienced over a lifetime increased. The findings are quite distributed and include gray and WM findings. Thus, in chronic mTBI, more simple dichotomous groupings into lifetime presence or absence of mTBI results will result in a potential bias toward the null and therefore possibly misleading non-significant findings.

Perhaps, there are a couple of important points that can be made with the current results. Compared to other clinical groupings, those reporting blast to be the primary mechanism of their signature mTBI had a mix of significant and trend level differences in the central WM volumetric and diffusion findings (mainly the corpus callosum). The differences were in expected directions indicating possible injury or degeneration. In contrast, veterans and SMs with multiple exposures (regardless of mechanism) had distributed volumetric and diffusion findings in medial temporal structures and cortical surfaces but not always in the expected direction (i.e., hypertrophy). The more central WM pattern of blast-related findings and the more widely distributed cortical surface and medial temporal lobe findings might be expected as animal and human studies show that the pathological and imaging findings in the corpus callosum are quite common following blast exposures^38^ and include pathological changes in myelin that impact size and shape of the axons in this area. Associations which might be labeled as unexpected (i.e., hypertrophy of hippocampus) are more difficult to explain but not completely unanticipated given that there are studies that have demonstrated similar findings. For example, Ross et al.^39^ observed hypertrophy in some regional volumetric measures in a subset of TBI patients following mild-to-moderate TBI and Govola et al.^40^ found Mossy cell hypertrophy within the hippocampus that correlated with significant increases in microglial activity and subsequent volumetric differences. Furthermore, it appears that sex may also play a role in the findings for this particular sample with the majority of the hypertrophy being associated with women in this sample. However, more research will be required to fully understand these findings, especially in chronic samples.

There are a limited number of studies that have examined imaging findings more than 1 year post-injury so that the chronic effects of mTBI on MRI imaging have not yet been fully elucidated. The length of time since signature injury was included as a covariate in the current analysis and suggests that a signal in the imaging data is identifiable even at longer TSI time frames. The lack of prior studies with clear information about the events between the time of injury and the assessment (i.e., rehabilitation, treatment regimes, additional injuries, mental health history, etc.) highlights the need for additional studies that examine longer post-injury durations and many potential moderating factors. Regardless, an association is clearly observable and should be acknowledged even though it is expected that there will be important variables yet to be identified that will modify typical brain structure aging trajectories.

There a several limitations that should be noted. First, as discussed earlier, the sample used in this research included only chronic mTBI. There is currently a limited amount of clinical information (i.e., rehabilitation, medications, etc.) available for the interval between the injury and baseline assessment, and there may be several factors that could impact findings in a way not yet understood. In addition, the findings associated with sex differences also require additional investigation and research in samples that have a more equivalent distribution of sex. Second, the fact that this study focuses on mTBI limits the generalizability beyond mTBI. In fact, even “complicated” mTBI or mTBI accompanied by day of injury CT/MRI findings would have been excluded because DoD/Veterans Affairs criterion were used to classify participants and any day of injury imaging findings would have eliminated these participants from inclusion. Third, this is primarily a descriptive study and there are additional demographic and clinical variables not considered in these analyses including comorbid clinical measures of symptom severity (i.e., headaches and post-concussive symptoms) or even cognitive function (i.e., neuropsychological performance). We have limited the number of potential clinical variables included in these initial analyses because of the effects that this has on statistical power when including multiple clinical variables in more traditional statistical methods but future research with more robust methods will be considered. However, we have also relied on these statistical methods so that the interpretability remains more direct and straightforward as more advanced methods sometimes make it more difficult to understand the clinical implications of the results.

## CONCLUSIONS

This study uses the growing CENC/LIMBIC observational cohort of SMs and veterans to examine the relationship between mTBI characteristics and neuroimaging findings. The findings were limited when simple dichotomous groupings were compared (i.e., no TBI history with mTBI history). When examining specific injury attributes, we identified structural brain changes in those who have suffered a blast-related TBI and/or when they experienced multiple mTBIs over their lifespan. These findings emphasize the need to proceed with more refined analyses that consider clinical aspects such as mechanism of injury and number of brain injuries. Even in studies with sufficient power, such as the present study, the subtlety and distributed nature of the injury across the brain of individuals in the group may obfuscate potential imaging findings. Future studies should focus on methods that might be able to identify patients with overlapping imaging information (i.e., imaging phenotypes) that can be used to categorize the individual patient in more clinically meaningful ways.

## Supplementary Material

usae031_Supp

## Data Availability

The data that support the findings in this study are publicly available through the Federal Interagency Traumatic Brain Injury Registry repository.
