# Supplementary material for: Persistent MRI Findings Unique to Blast and Repetitive Mild TBI: Analysis of the CENC/LIMBIC Cohort Injury Characteristics
Source: Mil Med. 2024 Feb 24;189(9-10):e1938–46. doi: 10.1093/milmed/usae031 (PMC11363162; doi:10.1093/milmed/usae031)
Supplement: usae031_Supp [file usae031_supp.zip › Table_S1_Interactions_with_sex.docx]

| **Table S1. Regional associations with mTBI count moderated by sex.** | | | |
| --- | --- | --- | --- |
| **Region** | **B** | **Z** | **p** |
| rh_inferiorparietal_thickness | -0.24 | -2.84 | p<0.01 |
| rh_parstriangularis_thickness | -0.23 | -3.23 | p<0.01 |
| rh_postcentral_thickness | -0.22 | -2.90 | p<0.01 |
| rh_rostralmiddlefrontal_thickness | -0.21 | -2.82 | p<0.01 |
| rh_superiorfrontal_thickness | -0.27 | -3.41 | p<0.01 |
| rh_superiorparietal_thickness | -0.21 | -2.75 | p<0.01 |
| lh_parstriangularis_thickness | -0.28 | -3.87 | p<0.01 |
| lh_posteriorcingulate_thickness | -0.24 | -3.06 | p<0.01 |
| lh_superiorfrontal_thickness | -0.28 | -3.41 | p<0.01 |
| ACR_R_FA | -0.28 | -2.50 | p<0.05 |
| CR_R_FA | -0.38 | -4.08 | p<0.01 |
| PCR_R_FA | -0.24 | -2.72 | p<0.01 |
| SCR_R_FA | -0.32 | -3.36 | p<0.01 |
| EC_R_FA | -0.39 | -3.12 | p<0.01 |
| PTR_R_FA | -0.30 | -2.63 | p<0.01 |
| UNC_R_FA | -0.36 | -4.45 | p<0.01 |
| ACR_L_FA | -0.24 | -2.77 | p<0.01 |
| PCR_L_FA | -0.25 | -2.74 | p<0.01 |
| CST_L_FA | 0.25 | 2.40 | p<0.05 |
| EC_L_FA | -0.48 | -4.08 | p<0.01 |
| PTR_L_FA | -0.23 | -2.56 | p<0.05 |
| SLF_L_FA | -0.23 | -2.35 | p<0.05 |
| UNC_L_FA | -0.49 | -5.18 | p<0.01 |
| ACR_FA | -0.29 | -3.12 | p<0.01 |
| ALIC_FA | -0.27 | -2.26 | p<0.05 |
| Average_FA | -0.25 | -2.37 | p<0.05 |
| CC_FA | -0.29 | -2.56 | p<0.05 |
| GCC_FA | -0.31 | -2.61 | p<0.01 |
| CR_FA | -0.28 | -2.97 | p<0.01 |
| EC_FA | -0.39 | -3.97 | p<0.01 |
| FX_FA | -0.33 | -3.03 | p<0.01 |
| IFO_FA | -0.26 | -2.55 | p<0.05 |
| PTR_FA | -0.31 | -2.99 | p<0.01 |
| SLF_FA | -0.23 | -2.30 | p<0.05 |
| UNC_FA | -0.37 | -4.50 | p<0.01 |
| UNC_R_RD | 0.37 | 4.31 | p<0.01 |
| UNC_RD | 0.47 | 4.93 | p<0.01 |
| CGH_L_AD | -0.30 | -3.42 | p<0.01 |
| Reported regions varied in association with mTBI count based on participant sex. Statistics for the sex-by-region interaction terms in each model are provided. | | | |
